# Associations between leaderboard usage in physical activity apps and perceived stress among university students: the roles of social comparison and physical activity

**DOI:** 10.3389/fpubh.2026.1794299

**Published:** 2026-05-14

**Authors:** Sha Wang, Mengyuan Zhang, Sudawan Wutichat, Theeranan Tanphanich, Hansen Li, Xing Zhang

**Affiliations:** 1 Physical Education College, Xichang University, Xichang, China; 2 Faculty of Education and Development Sciences, Kasetsart University Kamphaeng Saen Campus, Nakhon Pathom, Thailand; 3 School of Physical Education, Sichuan Agricultural University, Ya'an, China; 4 Institute of Sports Science, College of Physical Education Southwest University, Chongqing, China; 5 Department of Physical Education and Sports, Faculty of Sport Sciences, University of Granada, Granada, Spain

**Keywords:** digital health, exercise, mental health, sports health, stress

## Abstract

Leaderboards are a common design element in smartphone-based health and physical activity tracking apps and embedded features and are widely used by young people. Although researchers have become increasingly interested in their behavioral implications, evidence remains limited regarding whether and how leaderboard usage relates to psychological outcomes. To address this gap, we conducted a cross-sectional survey involving 1,019 university students. Using structural equation modeling, we examined the associations between leaderboard usage, leaderboard-based ability social comparison, moderate-intensity physical activity, and perceived stress. Leaderboard usage was positively associated with social comparison (*β* = 0.298) and physical activity (*β* = 0.322), and social comparison was positively associated with physical activity (*β* = 0.201). Physical activity was negatively associated with perceived stress (*β* = −0.325). Leaderboard usage was also indirectly associated with higher physical activity via social comparison (*β* = 0.060). Although leaderboard usage showed no direct association with perceived stress (*β* = 0.004), it was indirectly associated with lower perceived stress through physical activity alone (*β* = −0.105) and through a serial pathway involving social comparison and physical activity (*β* = −0.019). The total association between leaderboard usage and perceived stress was negative (*β* = −0.120). Overall, these findings suggest that leaderboard usage may be associated with lower perceived stress among university students primarily through activity-related pathways.

## 1 Introduction

Engaging in regular physical activity confers a wide range of physical and psychological health benefits (1, 2). Extensive evidence shows that higher physical activity levels are associated with lower risks of cardiovascular disease and metabolic disorders, reduced premature mortality, and better mental health (1–6). Accordingly, promoting and maintaining physical activity has long been a key priority in public health and preventive medicine. Despite these well-established benefits, global physical activity levels have declined in recent decades (7). Pooled analyses of large population-based surveys indicate that a substantial proportion of people do not meet recommended activity guidelines, with adolescents and young adults at particularly high risk of insufficient physical activity (7, 8). Academic demands, increasingly sedentary routines, and lifestyle transitions during late adolescence and early adulthood may jointly contribute to this pattern, underscoring the need for effective and scalable approaches to support physical activity engagement (9, 10).

In recent years, the use of smartphone-based health and physical activity tracking apps and embedded features has increased rapidly worldwide (11). These digital tools commonly provide users with real-time feedback on activity levels, goal attainment, and progress over time, and are frequently promoted as promising means of encouraging physical activity (12). Among the various design elements in such apps, leaderboards have emerged as one of the most prevalent forms of gamification (13). Leaderboards display users’ performance rankings relative to others and have been regarded as potentially useful tools for promoting physical activity (13, 14). For example, Hydari, Adjerid (14) found that participation in Fitbit leaderboards was associated with an average increase of approximately 370 steps/day, although effects varied substantially across users. Yang and Koenigstorfer (13) further suggested that the effects of leaderboards on physical activity may depend on users’ relative rank. Specifically, users positioned near the top or bottom of a leaderboard reported stronger physical activity intentions than those in middle positions. However, existing research has focused primarily on whether leaderboards relate to physical activity, whereas comparatively less attention has been paid to the mechanisms underlying these associations and their potential psychological consequences.

Social comparison refers to the process by which individuals evaluate their own performance or abilities in relation to others (15, 16). In leaderboard contexts, social comparison may be particularly relevant because leaderboards explicitly rank users and may encourage them to assess their own performance against that of peers or significant others (13, 14). Through this process, leaderboard usage may be associated with physical activity both directly and indirectly through comparison-based motivation and self-evaluation. In addition, because physical activity confers a wide range of physical and psychological health benefits (1, 2), it is important to examine whether any association between leaderboard usage and physical activity may also extend to downstream outcomes such as perceived stress. These questions may be particularly relevant among university students, a population in which insufficient physical activity is common and digital technologies and app-based tools are highly embedded in daily life (9, 10, 17). Therefore, the present study used structural equation modeling (SEM) to examine whether leaderboard usage is associated with physical activity via social comparison and whether leaderboard usage is indirectly associated with perceived stress through physical activity among university students. The theoretical framework and hypotheses are presented in Section 2.

## 2 Theoretical framework and hypotheses

First, drawing on social comparison theory, frequent exposure to leaderboards is expected to increase users’ engagement in ability-based social comparison (18). Social comparison theory suggests that individuals are more likely to evaluate themselves against others when comparative information is salient and readily available (18). In health and physical activity tracking apps/features, leaderboards explicitly rank users’ activity performance, making relative standing visible and encouraging comparisons with peers or significant others (18, 19). Accordingly, we hypothesized that leaderboard usage would be positively associated with leaderboard-based ability social comparison (hereafter referred to as social comparison) (H1).

Second, from self-regulatory and goal-setting perspectives, leaderboard usage may be positively associated with physical activity because it makes performance feedback salient and provides clear standards against which users can evaluate their current standing (20). By highlighting discrepancies between one’s present performance and a desired or higher-ranked position, leaderboards may motivate users to regulate their behavior, such as by striving to improve their standing or keep pace with others (21, 22). Accordingly, we hypothesized that leaderboard usage would be positively associated with physical activity (H2).

Third, social comparison may further shape physical activity behavior through self-evaluative and motivational processes (23). Consistent with social comparison theory, comparing one’s performance with that of others may prompt individuals to adopt higher standards, increase effort, or attempt to reduce perceived performance gaps (20, 24). From a self-regulatory perspective, these comparison-based discrepancies may motivate behavioral adjustment and greater goal-directed effort (23). In activity-tracking contexts, social-comparison cues have been associated with increases in physical activity in some studies, particularly when they provide interpretable performance feedback or competitive challenge (19, 21, 25). Such processes may be especially relevant in leaderboard contexts, where performance differences are explicitly displayed. Accordingly, we hypothesized that social comparison would be positively associated with physical activity (H3).

Fourth, consistent with extensive evidence on the mental health benefits of physical activity, higher levels of physical activity were expected to be associated with lower perceived stress (26). Physical activity may alleviate stress through physiological pathways (e.g., neuroendocrine regulation) and psychological mechanisms (e.g., improved mood and perceived competence) (27–29). Thus, we hypothesized a negative association between physical activity and perceived stress (H4).

Taken together, based on the above social comparison and self-regulatory mechanisms, we hypothesized that leaderboard usage would be indirectly associated with lower perceived stress through two routes: (a) an indirect pathway via physical activity alone (H5), and (b) a serial mediation pathway involving social comparison and physical activity (H6). Given limited evidence on the direct association between leaderboard usage and perceived stress, we did not specify an *a priori* directional hypothesis for this path. In all models, gender and educational stage (year of study) were included as covariates because prior research indicates that physical activity and related behavioral patterns differ across gender and study-stage characteristics in university student populations (9, 10). These demographic characteristics may also be associated with perceived stress and were therefore controlled for in the analyses (30, 31). The proposed theoretical model is presented in Supplementary Figure 1.

## 3 Methods

### 3.1 Study design and participants

A cross-sectional survey was administered between January 1 and January 10, 2026. The survey included preexisting standardized measures as well as adapted and study-developed items. Specifically, perceived stress was assessed using the Chinese version of the 10-item Perceived Stress Scale (PSS-10), moderate-intensity physical activity using the International Physical Activity Questionnaire–Short Form (IPAQ-SF), leaderboard-based ability social comparison using an adapted prior measure, and leaderboard usage using a brief study-developed instrument. The adapted and study-developed items were refined through pilot testing. The final survey was hosted on Sojump^1^, a widely used online platform in China for questionnaire design, distribution, and data collection (32). To disseminate the survey, we recruited 15 university teachers from diverse academic disciplines, who shared a quick response (QR) code linking to the survey in their online course chat groups. During recruitment, the survey was presented as a study of university students’ use of health and physical activity tracking apps/features; participants were not informed of the specific study hypotheses to minimize response bias. Participation was voluntary and uncompensated, and participants could withdraw at any time. All participants provided electronic informed consent before starting the survey. The study was conducted in accordance with the Declaration of Helsinki and was approved by the Ethics Review Committee of Southwest University (SWUETH2024307007).

Eligibility criteria were: (1) being a university student; and (2) having prior experience using leaderboard functions in health or physical activity tracking apps/features. Responses were excluded if (1) the questionnaire was incomplete, or (2) the respondent failed data-quality verification checks intended to ensure attentive responding and to exclude responses likely generated by automated systems, random entries, or other invalid patterns. To prevent duplicate participation, access restrictions were implemented based on device, account, and IP address information.

### 3.2 Instruments and measurements

#### 3.2.1 Stress

Perceived stress was measured using the Chinese version of the PSS-10 (33). Respondents indicated how often they experienced stress-related thoughts and feelings during the past month on a 5-point scale (0 = never to 4 = very often). Four positively worded items were reverse-coded prior to score calculation. Total scores range from 0 to 40, with higher scores indicating greater perceived stress. Internal consistency in the current sample was acceptable (Cronbach’s *α* = 0.75).

#### 3.2.2 Leaderboard-based ability social comparison

Social comparison was assessed using a context-specific measure adapted from the Social Media Social Comparison of Ability measure reported by Yang, Holden (34). Items were reworded to reflect health and physical activity tracking apps/features with leaderboard functions (e.g., step counts, workout frequency, pace, and distance) and were introduced with the stem: “During the past 7 days, when viewing rankings/leaderboards in smartphone health or physical activity tracking apps….” The adapted scale comprised five items assessing comparisons with other users, monitoring one’s relative standing, using others as a benchmark for self-evaluation, and comparisons with significant others. One item was reverse-coded (“I generally do not use leaderboards to compare myself with others to evaluate my exercise/training performance”). Responses were made on a 5-point scale (1 = not at all, 5 = very often). After reverse-coding, item scores were summed to yield a total score (range = 5–25), with higher scores indicating more frequent ability-based social comparison in leaderboard contexts. Internal consistency in the current sample was acceptable (Cronbach’s *α* = 0.91).

#### 3.2.3 Physical activity

Physical activity was assessed using the International Physical Activity Questionnaire–Short Form (IPAQ-SF) (35). The main analysis focused on moderate-intensity physical activity, calculated as MET-min/week = 4.0 × minutes/day × days/week, following the IPAQ scoring protocol. Supplementary analyses were also conducted using vigorous-intensity physical activity and combined moderate-to-vigorous physical activity (MVPA), and these results are reported in Supplementary Tables 1, 2.

#### 3.2.4 Leaderboard usage

Leaderboard usage in health and physical activity tracking apps/features was assessed using a brief, study-developed instrument. Participants were first screened for whether they had used any leaderboard/ranking/challenge-board function in health or physical activity tracking apps/features. Only those who reported such leaderboard use were then asked the subsequent leaderboard questions. Specifically, participants reported: (1) the number of days during the past 7 days on which they viewed any leaderboard/ranking/challenge-board pages within health and physical activity tracking apps/features (e.g., WeChat step-count leaderboards; Keep rankings/challenges; Strava leaderboards; Huawei Health or Mi Fitness rankings/challenges); and (2) average daily checking frequency on leaderboard-use days, coded as 1 (“1 time/day”), 2 (“2–3 times/day”), 3 (“4–6 times/day”), and 4 (“≥ 7 times/day”). A composite leaderboard usage score was calculated as the product of the two item scores, with higher scores indicating more frequent leaderboard use during the past week.

### 3.3 Statistical analysis

Demographic characteristics and study variables were summarized as means and standard deviations (SD) for continuous variables and as counts with percentages for categorical variables. Given the non-normal distributions of key variables, Spearman’s rank-order correlations were used to examine bivariate associations. Correlation magnitudes were interpreted using Cohen’s guidelines (based on |*ρ*|), with values of approximately 0.10, 0.30, and 0.50 indicating small, medium, and large associations, respectively (36). To test the hypothesized directional relationships in the conceptual framework, structural equation modeling (SEM) was conducted. Following Bagozzi and Yi (37), the sample size (*N* = 1,019) exceeded the recommended minimum relative to the number of free parameters. Potential multicollinearity among predictors was assessed using variance inflation factors (VIFs), with values <5 indicating no evidence of serious multicollinearity (38). Models were estimated using maximum likelihood estimation. To obtain robust standard errors and confidence intervals for all path estimates, bias-corrected bootstrapping with 10,000 resamples was applied (39–41). Model fit was evaluated using standard global fit indices, including χ^2^/df, the comparative fit index (CFI), goodness-of-fit index (GFI), adjusted goodness-of-fit index (AGFI), normed fit index (NFI), and relative fit index (RFI). Mediation was evaluated via indirect effects (i.e., the product of the constituent path coefficients), which were considered statistically significant when the 95% bias-corrected bootstrap confidence interval excluded zero (42, 43). All analyses were conducted using SPSS 26.0 and AMOS 24.0, with two-sided *p* < 0.05 indicating statistical significance (44, 45). There were no missing data in the final analytic sample because incomplete questionnaires were excluded prior to analysis.

Supplementary SEM analyses were additionally conducted using vigorous-intensity physical activity and combined MVPA as alternative behavioral outcomes to examine the robustness of the findings across physical activity intensity domains.

### 3.4 Alternative models

To examine the robustness of the SEM findings, we specified and compared three theoretically plausible alternative models. Alternative Model 1 (stress-driven model) treated perceived stress as the antecedent (X) and leaderboard usage as the outcome (Y) to test whether the reverse directional ordering could also account for the observed associations, while retaining the remaining structural relations to form a coherent stress-driven pathway. Alternative Model 2 (additional direct path) added a direct path from social comparison to perceived stress (social comparison → stress) to evaluate whether social comparison is associated with stress beyond the proposed mediation pathway. Alternative Model 3 (alternative mediator ordering) tested the specificity of the mediator sequence by re-specifying the serial mediation chain as leaderboard usage → physical activity → social comparison → stress. Detailed model specifications are provided in Supplementary Figure 2. Model comparisons were based on information criteria (AIC and BIC), with lower values indicating a better balance between model fit and parsimony.

## 4 Results

### 4.1 Characteristics of participants

A total of 1,019 university students were included. Overall, the sample was predominantly aged 17–20 years and mainly comprised freshman and sophomore students. Most participants reported engaging in moderate-intensity physical activity 1–3 days per week. Detailed participant characteristics are presented in Table 1.

**Table 1:** Characteristics of the participants.

Variable	Category	*N*	Percentage
Gender	Male	566	55.5%
	Female	453	44.5%
Age (year)	17–20	908	89.1%
	21–23	93	9.1%
	>23	18	1.8%
Educational stage	Freshman student	595	58.4%
	Sophomore student	344	33.8%
	Junior student	42	4.1%
	Senior student	16	1.6%
	Graduate student	22	2.2%
Moderate-intensity physical activity	None	114	11.2%
	1–3 days/week	663	65.1%
	4–6 days/week	161	15.8%
	Every day	81	7.9%
		Mean	SD
Stress (PSS-10 score)		14.87	6.00
Social comparison (score)		15.12	3.76
Moderate-intensity physical activity (MET-min/week)		724.91	1062.81
Leaderboard usage (score)		5.45	7.69

PSS-10, 10-item Perceived Stress Scale; SD, standard deviation.

### 4.2 Correlation analysis

**Figure 1:**
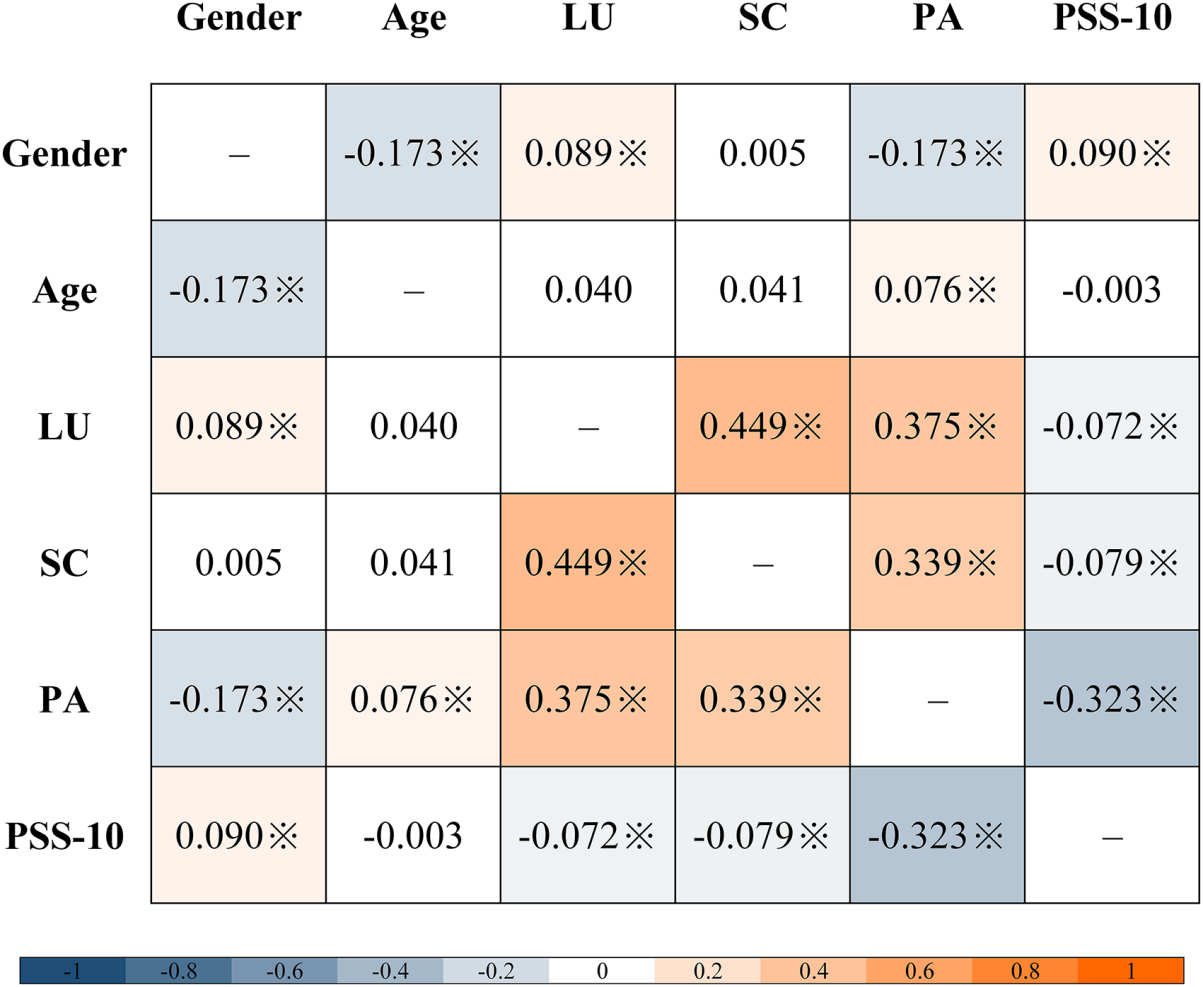
Correlation heatmap (※ *p* < 0.05, two-tailed; LU, leaderboard usage; SC, social comparison; PA, moderate-intensity physical activity; PSS-10, perceived stress).

Spearman’s rank-order correlations (Figure 1) indicated that leaderboard usage was moderately positively correlated with social comparison (*ρ* = 0.449) and physical activity (ρ = 0.375). Social comparison was also moderately positively correlated with physical activity (ρ = 0.339). By contrast, perceived stress was moderately negatively correlated with physical activity (ρ = −0.323) and weakly negatively correlated with leaderboard usage (ρ = −0.072) and social comparison (ρ = −0.079).

### 4.3 Structural equation modeling analysis

#### 4.3.1 Model comparison

The hypothesized model yielded the lowest information criteria (AIC = 36.733; BIC = 125.411), compared with Alternative Model 1 (AIC = 80.915; BIC = 169.593), Alternative Model 2 (AIC = 38.502; BIC = 132.107), and Alternative Model 3 (AIC = 165.232; BIC = 253.913). Given that lower AIC and BIC values indicate a better balance between model fit and parsimony, the hypothesized model was retained as the final model.

#### 4.3.2 Total, direct, and indirect associations

**Figure 2:**
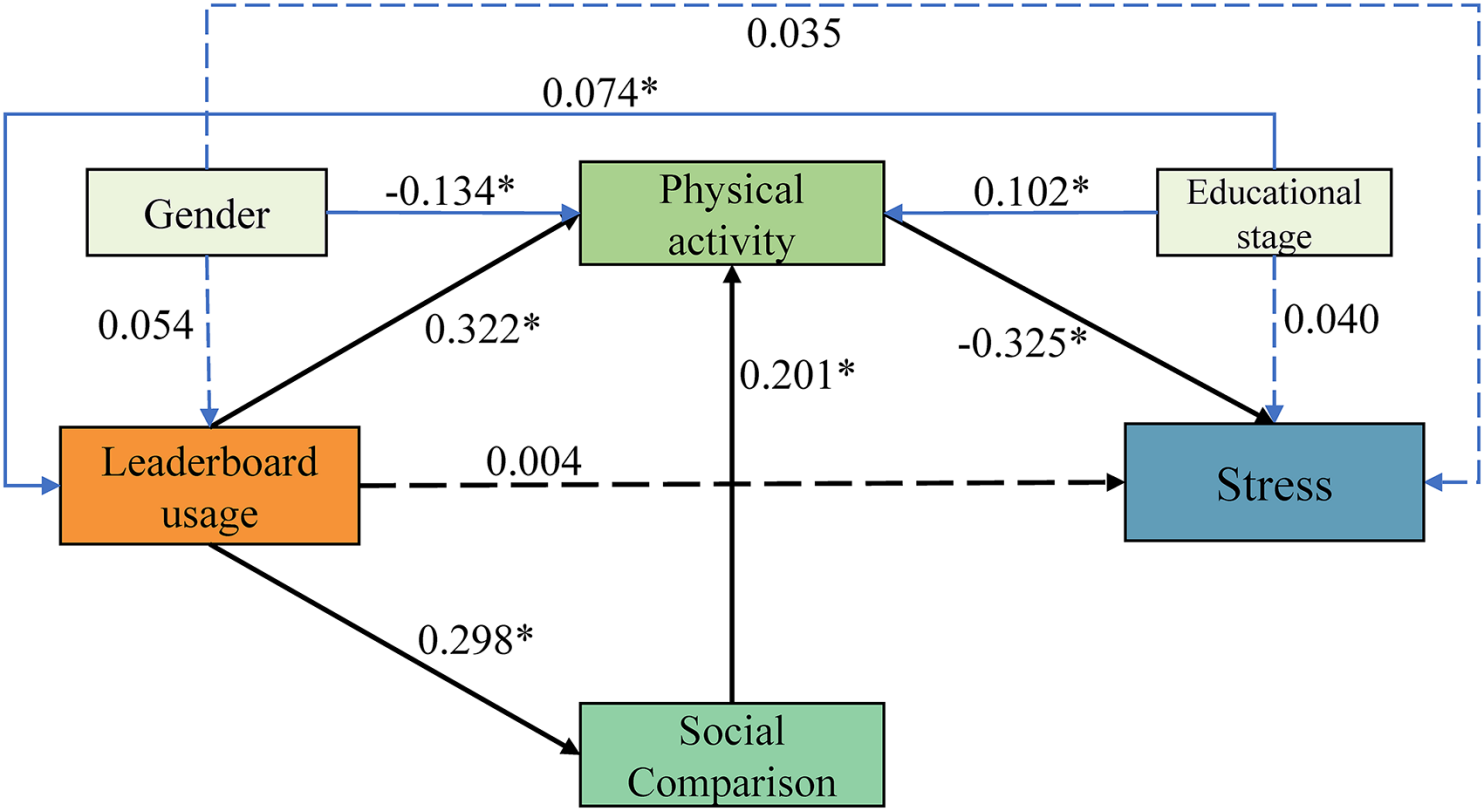
The results of path analysis (*, *p* < 0.05).

As shown in Table 2 and Figure 2, leaderboard usage was positively associated with social comparison (*β* = 0.298, 95% CI = 0.225 to 0.362, *p* < 0.001) and physical activity (*β* = 0.322, 95% CI = 0.241 to 0.395, *p* < 0.001). Social comparison was also positively associated with physical activity (*β* = 0.201, 95% CI = 0.154 to 0.245, *p* < 0.001), whereas physical activity was negatively associated with perceived stress (*β* = −0.325, 95% CI = −0.400 to −0.250, *p* < 0.001). In addition, leaderboard usage showed a significant indirect association with physical activity via social comparison (*β* = 0.060, 95% CI = 0.045 to 0.077, *p* < 0.001), and significant indirect associations with perceived stress via physical activity alone (*β* = −0.105, 95% CI = −0.143 to −0.070, *p* < 0.001) and via the serial pathway through social comparison and physical activity (*β* = −0.019, 95% CI = −0.027 to −0.013, *p* < 0.001). Social comparison was also indirectly associated with lower perceived stress via physical activity (*β* = −0.065, 95% CI = −0.089 to −0.045, *p* < 0.001). The direct association between leaderboard usage and perceived stress was not significant (*β* = 0.004, 95% CI = −0.061 to 0.069, *p* = 0.927), whereas the total association between leaderboard usage and perceived stress was negative (*β* = −0.120, 95% CI = −0.184 to −0.055, *p* = 0.001).

**Table 2:** The total, direct, and indirect associations.

Pathway	Standardized *β*	95% CI	*p*
Total association	−0.120	−0.184 to −0.055	0.001
LU → SC	0.298	0.225 to 0.362	<0.001
LU → PA	0.322	0.241 to 0.395	<0.001
LU → PSS-10	0.004	−0.061 to 0.069	0.927
SC → PA	0.201	0.154 to 0.245	<0.001
PA → PSS-10	−0.325	−0.400 to −0.250	<0.001
LU → SC → PA → PSS-10	−0.019	−0.027 to −0.013	<0.001
LU → SC → PA	0.060	0.045 to 0.077	<0.001
LU → PA → PSS-10	−0.105	−0.143 to −0.070	<0.001
SC → PA → PSS-10	−0.065	−0.089 to −0.045	<0.001

LU, leaderboard usage; SC, social comparison; PA, moderate-intensity physical activity; PSS-10, 10-item Perceived Stress Scale.

Supplementary analyses showed that the MVPA model yielded a pattern broadly similar to the main model, whereas in the vigorous-intensity model the path from social comparison to vigorous-intensity physical activity and the corresponding serial indirect association were not significant (Supplementary Tables 1, 2).

## 5 Discussion

The present study examined how leaderboard usage in health and physical activity tracking apps/features relates to perceived stress among university students and whether these associations operate through social comparison and physical activity. Our SEM results indicated that leaderboard usage was positively associated with social comparison and physical activity, and that physical activity was inversely associated with perceived stress. Importantly, leaderboard usage was not directly associated with perceived stress; rather, its overall association with lower perceived stress was explained by indirect pathways via physical activity alone and via a serial pathway through social comparison and physical activity. In addition, social comparison mediated the association between leaderboard usage and physical activity and was indirectly associated with lower perceived stress through physical activity.

Our findings showed that, among university students, leaderboard usage was positively associated with social comparison (H1) and with physical activity (H2), and that social comparison was also positively associated with physical activity (H3). Consistent with the significant indirect effect of leaderboard usage on physical activity via social comparison, these results suggest that, in this student sample, leaderboards may influence physical activity both directly and indirectly through social comparison processes. The direct positive association between leaderboard usage and physical activity is consistent with prior research. For example, Hydari, Adjerid (14) found that participation in Fitbit leaderboards was associated with an increase of approximately 370 steps/day (≈3.5%), although effects varied substantially across users. One plausible explanation is that leaderboards provide university students with salient performance feedback and competitive cues, which may enhance self-monitoring, goal striving, and effort investment, thereby encouraging greater physical activity (21, 46). In addition, the positive association between leaderboard usage and social comparison is theoretically expected because leaderboards are designed to make relative standing salient by ranking users’ activity performance. Thus, more frequent leaderboard usage may increase students’ exposure to comparison cues and normative performance information, thereby prompting more frequent ability-based comparisons with peers or significant others (19).

The positive association between social comparison and physical activity among university students is broadly consistent with prior evidence suggesting that social-comparison cues in activity-tracking contexts can motivate behavior change. For example, Nastasi, Curry (25) reported that previously sedentary participants increased their step counts after being exposed to social-comparison information. One plausible explanation is that ability-based comparison may function primarily as an informational and motivational mechanism rather than a purely threatening experience (47). When university students observe others’ performance (or their own relative standing), they may set more ambitious goals, strengthen perceived norms around being active, or invest greater effort to reduce perceived performance gaps, which may translate into higher activity levels. However, the effects of social comparison are unlikely to be uniformly positive. Prior research suggests that upward or threatening comparisons may undermine self-evaluation and contribute to psychological distress in some contexts (47, 48). In leaderboard settings, repeated exposure to unfavorable comparison information, such as persistent low ranking or large performance gaps, may likewise be experienced less as a motivating cue and more as a psychological burden. Thus, future research should examine whether the effects of leaderboard-based social comparison depend on whether comparison information is perceived as motivating or threatening.

It is important to note that the effects of leaderboard usage on physical activity may be highly heterogeneous across individuals and design contexts. For example, Hydari, Adjerid (14) reported that highly active users (≥13,000 steps/day) exhibited decreases in daily step counts after starting to use leaderboard features (approximately −5%, or about −630 steps/day). The authors suggested that this negative effect may depend on leaderboard size: on smaller leaderboards, highly active users are more likely to be paired with—or primarily observe—less active peers, which may reduce competitive pressure and foster complacency, thereby lowering subsequent activity. Overall, this evidence suggests that leaderboards may not uniformly promote physical activity and that their effects likely depend on baseline activity levels and leaderboard characteristics (e.g., group size and peer composition). By contrast, we did not observe a negative association between leaderboard usage and physical activity in our university student sample. One plausible explanation is that overall activity levels were relatively low, with 76.3% of participants reporting moderate-intensity physical activity on 0–3 days/week—a profile for whom leaderboards may be more likely to be motivating than complacency-inducing. In addition, because the distribution of physical activity in our sample was highly skewed, we were unable to further examine whether baseline activity level moderated the direct and indirect associations in our model. Future studies could use sampling strategies to achieve more balanced activity distributions and test whether activity level and leaderboard design shape the leaderboard–physical activity relationship and its downstream consequences.

Based on our findings, physical activity was negatively associated with perceived stress among university students (H4). This pattern is consistent with a substantial body of evidence supporting the stress-buffering effects of physical activity (27–29). One plausible explanation is that regular activity may facilitate adaptive regulation of stress-related systems (e.g., autonomic and neuroendocrine functioning) and improve sleep and recovery (49). In addition, engaging in physical activity may enhance mood, perceived competence, and self-efficacy, while also providing a behavioral outlet for tension and a break from academic demands—mechanisms that may be particularly relevant in student populations (50, 51). Taken together with our earlier results showing that leaderboard usage was positively associated with physical activity among university students—both directly and indirectly via social comparison—these findings suggest a behavioral route through which leaderboard usage may be linked to lower perceived stress. However, we did not observe a significant direct association between leaderboard usage and perceived stress. Instead, the association appeared to operate primarily through indirect pathways, with the indirect effect via physical activity alone being larger (H5) than the serial pathway via social comparison and physical activity (H6). This pattern suggests that the stress-related association with leaderboard usage in our sample may be driven mainly by increased physical activity, whereas the additional contribution of social comparison—although statistically significant—was comparatively modest.

Notably, some prior evidence links social comparison to higher perceived stress and psychological distress (48, 52). This contrasts with our findings. Although our final model did not include a direct path from social comparison to perceived stress, Alternative Model 2—adding a direct social comparison → stress path—also showed no significant direct association. This discrepancy may reflect differences in the direction and valence of comparison: stress-related evidence often focuses on upward or threatening comparisons, whereas our measure captured the frequency of leaderboard-based comparison (48, 52). Context may also matter. Comparison in activity-tracking settings may function more as an informational or motivational cue that translates into higher physical activity, whereas comparison in appearance- or social-evaluation contexts may act as a stressor. Overall, among university students, leaderboard usage was positively associated with physical activity, both directly and indirectly via social comparison, and these activity-related pathways were associated with lower perceived stress. However, university students may not represent the most underserved group for physical activity promotion, and digital leaderboard-based approaches may not be equally effective across populations (53, 54). Future research should therefore examine whether similar associations are observed in more underserved populations.

## 6 Limitations

Several limitations should be acknowledged when interpreting our findings. First, our study focused on university students who had used leaderboard/ranking/challenge-board functions in health or physical activity tracking apps/features, which may limit the generalizability of the findings to other populations (e.g., working adults or older adults). In addition, although the main analysis focused on moderate-intensity physical activity, supplementary analyses using vigorous-intensity physical activity and combined MVPA suggested that some pathways were not fully consistent across intensity domains. Therefore, the findings should not be generalized uniformly across all physical activity intensity domains. Second, the study relied on self-reported measures, which may be subject to recall error and social desirability bias (55). Future research could incorporate more objective assessments, such as device-based tracking of physical activity and detailed logs of leaderboard usage. Third, recruitment via online chat groups may have introduced selection bias, as individuals who are less engaged in such groups may have been underrepresented (56). Future studies could mitigate this issue by adopting mixed recruitment strategies (e.g., combining online and offline approaches) and/or using stratified sampling to ensure more balanced samples. In addition, leaderboard usage was assessed as an overall cross-platform indicator rather than a platform-specific measure. During questionnaire development, several participants reported using leaderboard functions across multiple platforms, making it difficult to isolate the role of any single platform. Future research should distinguish between platform-specific leaderboard formats and examine whether their associations differ. Finally, our study employed a cross-sectional design, which restricts our ability to establish causal relationships between variables, although we examined the possibility of reverse causality using alternative model specifications (57). To further clarify causal pathways, future research should consider longitudinal designs or controlled experimental trials.

## 7 Conclusion

The current study examined how leaderboard usage in health and physical activity tracking apps/features relates to perceived stress among university students and whether these associations operate through social comparison and physical activity. Our findings indicate that, among university students who had used leaderboard/ranking/challenge-board functions in health or physical activity tracking apps/features, leaderboard usage was positively associated with moderate-intensity physical activity, both directly and indirectly via social comparison. In turn, higher physical activity levels were associated with lower perceived stress, suggesting that the overall association between leaderboard usage and perceived stress operates primarily through indirect pathways rather than a direct effect. Future longitudinal and experimental studies incorporating objective measures of physical activity and app usage are needed to clarify causal directions and to identify for whom—and under what conditions—leaderboard features support physical activity engagement and downstream psychological outcomes.

## Data Availability

The raw data supporting the conclusions of this article will be made available by the authors, without undue reservation.

## Footnotes

1
www.sojump.com
