# Supplementary material for: Associations between leaderboard usage in physical activity apps and perceived stress among university students: the roles of social comparison and physical activity
Source: Front Public Health. 2026 May 14;14:1794299. doi: 10.3389/fpubh.2026.1794299 (PMC13217397; doi:10.3389/fpubh.2026.1794299)
Supplement: Supplementary file 1 [file Supplementary_file_1.DOCX]

Supplementary Material


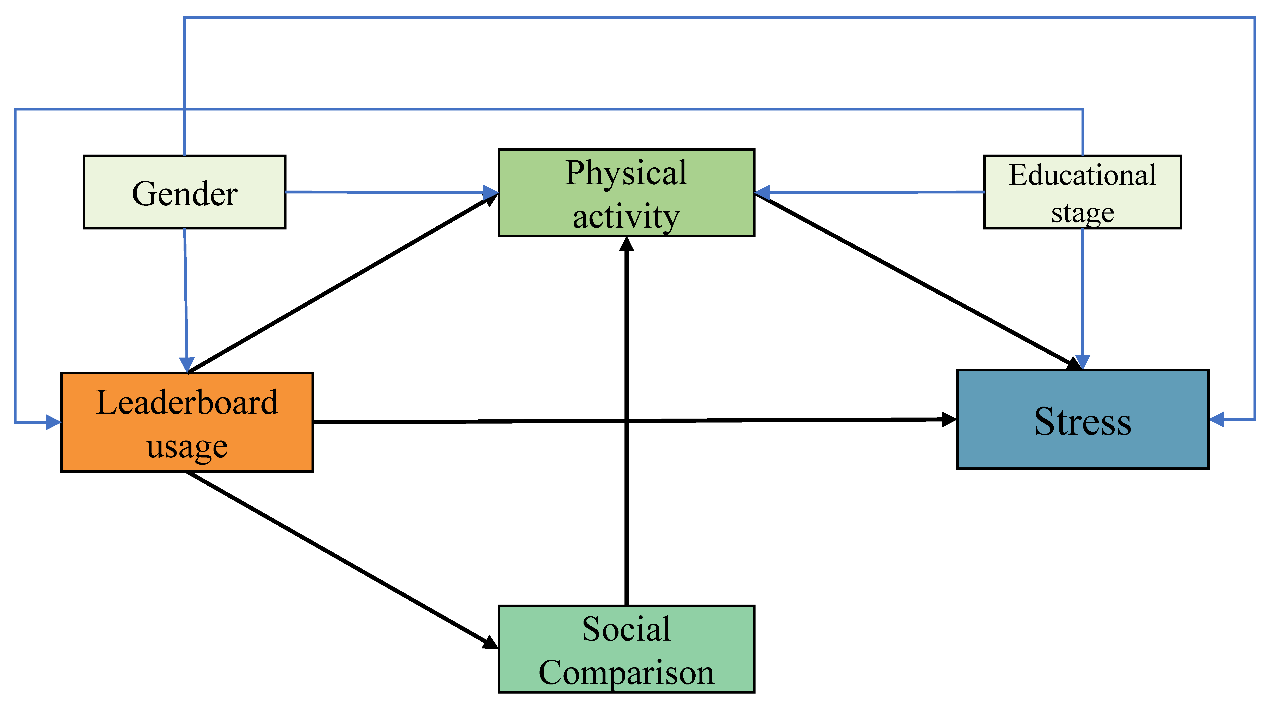


Supplementary Figure 1. Conceptual framework. Black lines indicate hypothesized pathways among the focal variables, and blue lines indicate covariate-adjustment paths from the control variables (gender and educational stage).


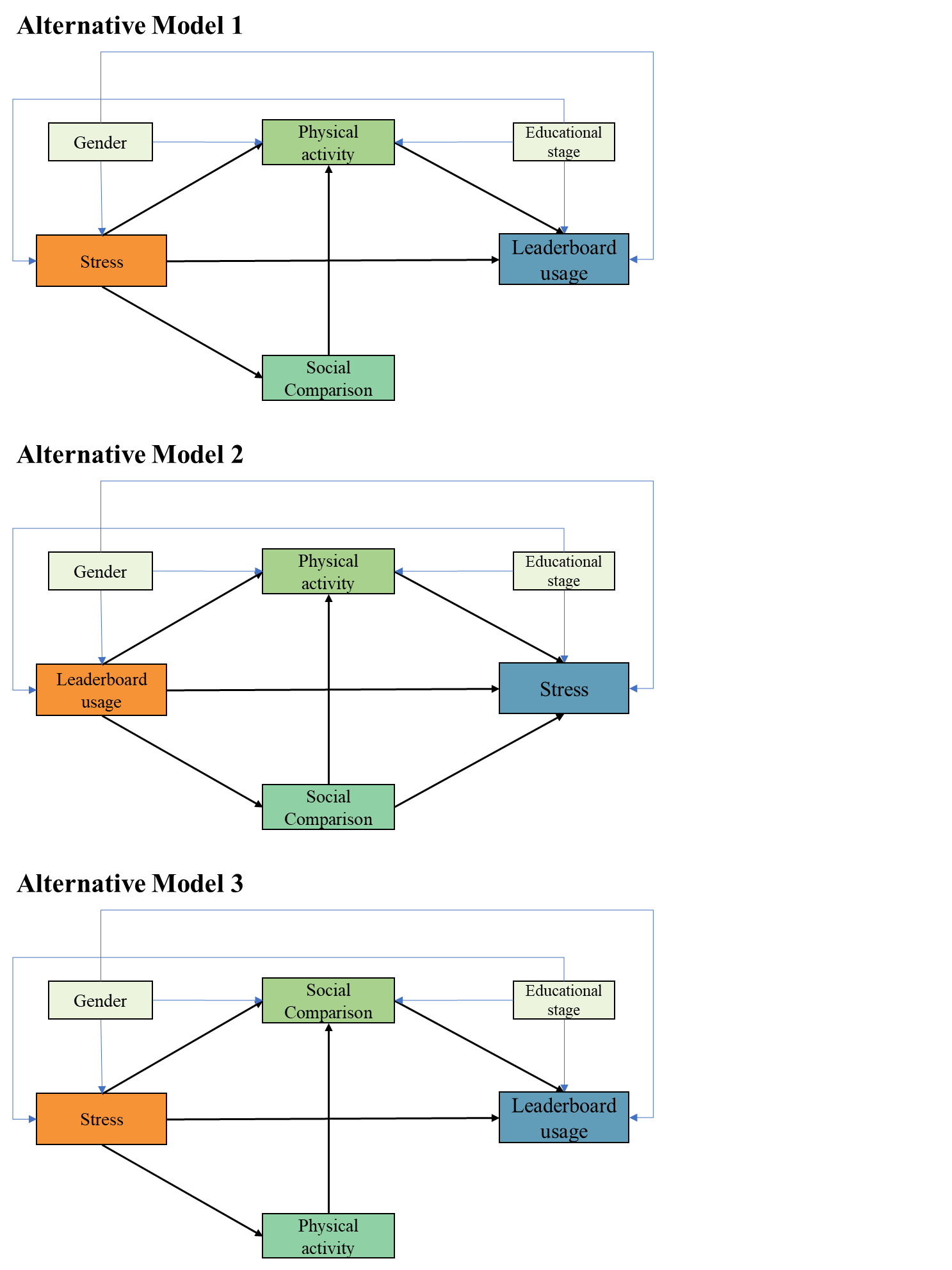


**Supplementary Figure 2.** Alternative models. Black lines indicate hypothesized pathways among the focal variables, and blue lines indicate covariate-adjustment paths from the control variables (gender and educational stage).

**Supplementary Table 1**. Direct, indirect, and total associations from the SEM model using vigorous-intensity physical activity (VPA) as the behavioral outcome

| **Pathway** | **Standardized β** | **95% CI** | **p** |
| --- | --- | --- | --- |
| **Direct associations** |  |  |  |
| Leaderboard usage → Social comparison | 0.298 | 0.226 to 0.364 | < 0.001 |
| Leaderboard usage → Vigorous-intensity physical activity | 0.378 | 0.306 to 0.444 | < 0.001 |
| Leaderboard usage → Perceived stress | -0.043 | -0.115 to 0.027 | 0.223 |
| Social comparison → Vigorous-intensity physical activity | 0.019 | -0.045 to 0.081 | 0.524 |
| Vigorous-intensity physical activity → Perceived stress | -0.143 | -0.222 to -0.069 | < 0.001 |
| **Indirect associations** |  |  |  |
| Leaderboard usage → Social comparison → Vigorous-intensity physical activity | 0.006 | -0.014 to 0.024 | 0.521 |
| Leaderboard usage → Vigorous-intensity physical activity → Perceived stress | -0.054 | -0.090 to -0.026 | < 0.001 |
| Leaderboard usage → Social comparison → Vigorous-intensity physical activity → Perceived stress | -0.001 | -0.004 to 0.002 | 0.462 |
| Social comparison → Vigorous-intensity physical activity → Perceived stress | -0.003 | -0.014 to 0.006 | 0.467 |
| **Summary associations** |  |  |  |
| Total indirect association: Leaderboard usage → Perceived stress | -0.055 | -0.092 to -0.026 | < 0.001 |
| Total association: Leaderboard usage → Perceived stress | -0.097 | -0.164 to -0.034 | 0.003 |

Note. Estimates are standardized coefficients (β). Leaderboard usage and social comparison were modeled as predictors, vigorous-intensity physical activity (VPA) as the behavioral mediator/outcome, and perceived stress as the final outcome. Gender and educational stage were included as covariates in the model but are not displayed here.

**Supplementary Table 2**. Direct, indirect, and total associations from the SEM model using moderate-to-vigorous physical activity (MVPA) as the behavioral outcome

| **Pathway** | **Standardized β** | **95% CI** | **p** |
| --- | --- | --- | --- |
| **Direct associations** |  |  |  |
| Leaderboard usage → Social comparison | 0.298 | 0.226 to 0.364 | < 0.001 |
| Leaderboard usage → Physical activity | 0.395 | 0.329 to 0.455 | < 0.001 |
| Leaderboard usage → Perceived stress | 0.004 | -0.067 to 0.073 | 0.901 |
| Social comparison → Physical activity | 0.108 | 0.055 to 0.160 | < 0.001 |
| Physical activity → Perceived stress | -0.256 | -0.332 to -0.180 | < 0.001 |
| **Indirect associations** |  |  |  |
| Leaderboard usage → Social comparison → Physical activity | 0.032 | 0.017 to 0.050 | < 0.001 |
| Leaderboard usage → Physical activity → Perceived stress | -0.101 | -0.140 to -0.069 | < 0.001 |
| Leaderboard usage → Social comparison → Physical activity → Perceived stress | -0.008 | -0.014 to -0.004 | < 0.001 |
| Social comparison → Physical activity → Perceived stress | -0.028 | -0.046 to -0.013 | < 0.001 |
| **Summary associations** |  |  |  |
| Total indirect association: Leaderboard usage → Perceived stress | -0.109 | -0.151 to -0.076 | < 0.001 |
| Total association: Leaderboard usage → Perceived stress | -0.105 | -0.171 to -0.041 | 0.001 |

Note. Estimates are standardized coefficients (β). Leaderboard usage and social comparison were modeled as predictors, moderate-to-vigorous physical activity (PA) as the behavioral mediator/outcome, and perceived stress as the final outcome. Gender and educational stage were included as covariates in the model but are not displayed here.
